# Supplementary material for: NF-κB and TNF Affect the Astrocytic Differentiation from Neural Stem Cells
Source: Cells. 2021 Apr 8;10(4):840. doi: 10.3390/cells10040840 (PMC8068246; doi:10.3390/cells10040840)
Supplement: Supplementary file 1 [file cells-10-00840-s001.pdf]

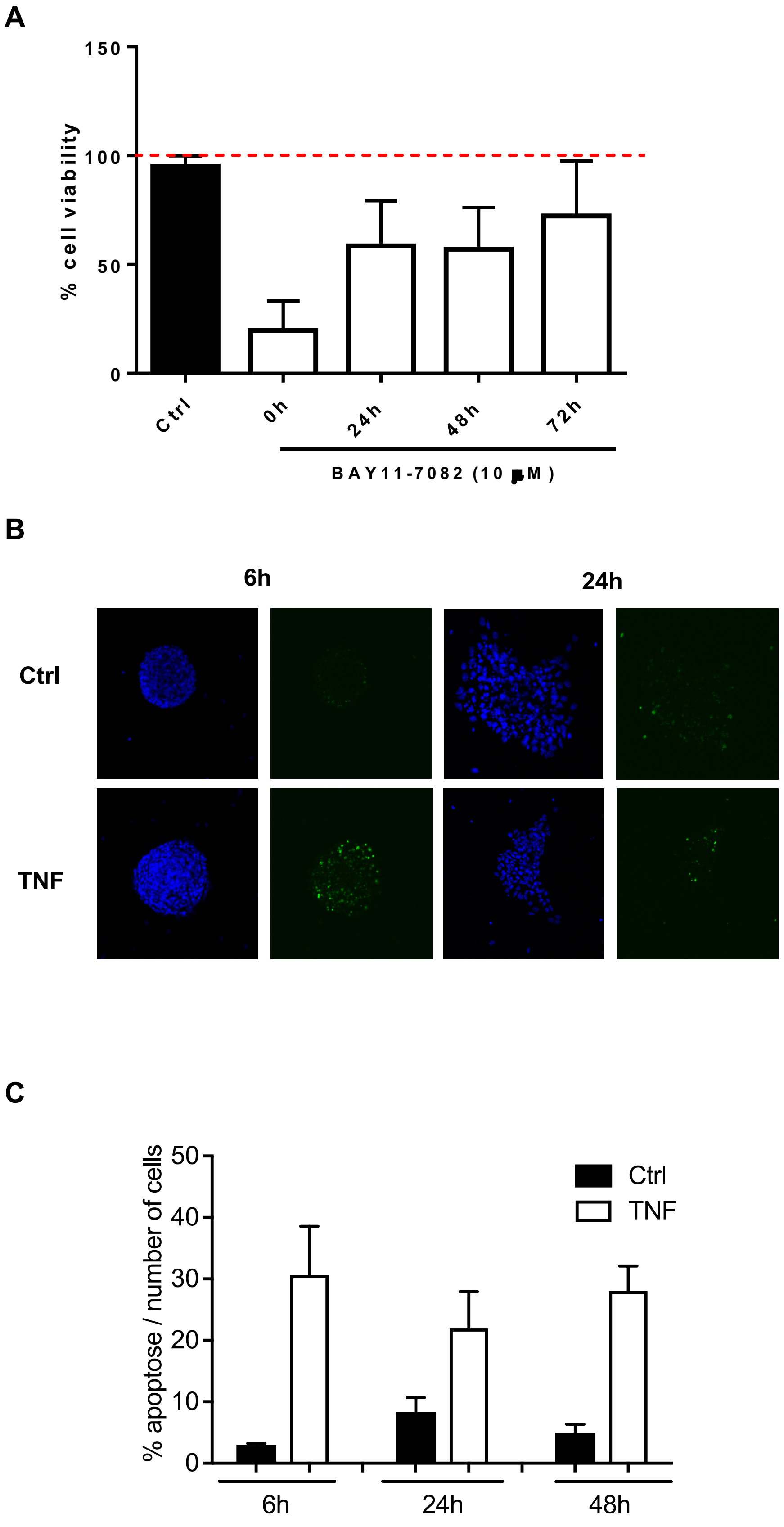

S1. A: Cell viability assessed by a MTT assay of NSP-derived astrocytes after 96h of differentiation with BAY11-7082 (mean±SEM). BAY11-7082 (10mM) was added at different time during astrocytic differentiation (0, 24, 48, 72h). B: TUNEL assay measuring TNF-induced cell death. Nuclei were stained with DAPI (blue) and TUNEL positive cells display green nuclei. Scale bar: 50 µm. C: Quantification of TUNEL positive cells for each condition (FCS, TNF) at 6, 24, and 48h. The percentage of TUNEL positive cells is represented relative to the total number of cells.

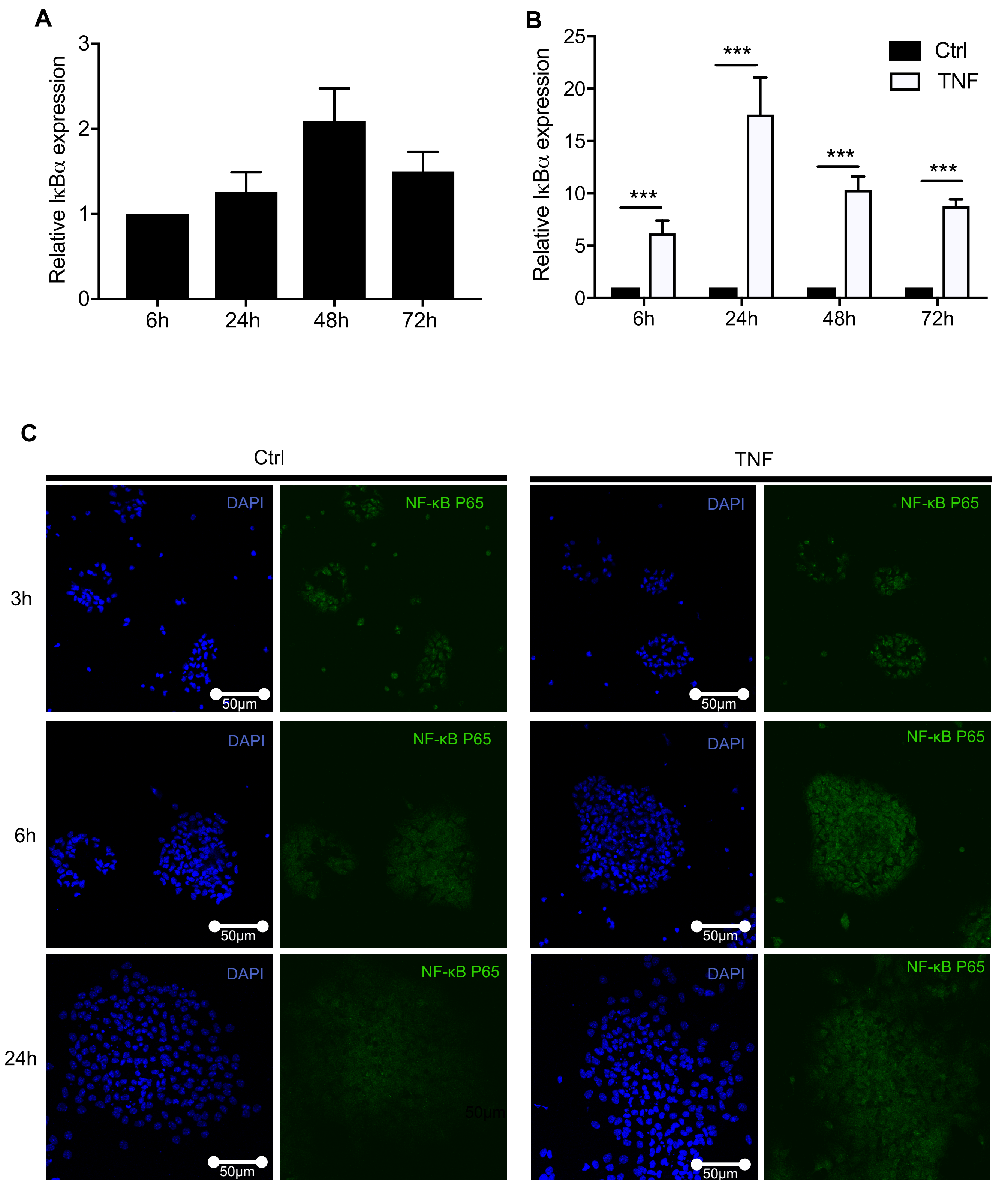

S2. A: kinetic of *IκBα* mRNA expression obtained by microarrays analyses (n=3). B: Activation of the NF-kB pathway by TNF. Effect of TNF treatment on *IκBα* gene expression obtained by RT-PCR. Each time point is normalized to its FBS control (=1). Results are given as mean ± SEM of 3 independent experiments. ***p<0,001. C: Immunocytochemistry showing NF-kB P65 protein expression (green) in TNF treated cells at 3, 6, and 24h. Nuclei were counterstained by DAPI (blue). Scale bar: 50 µm.

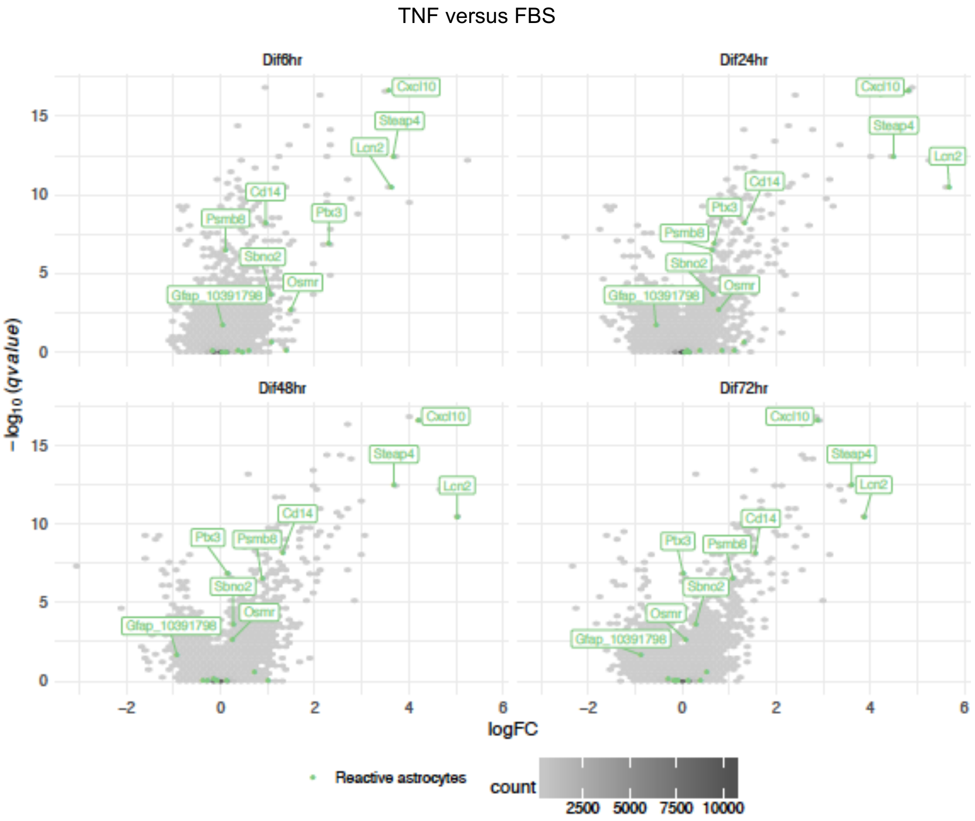

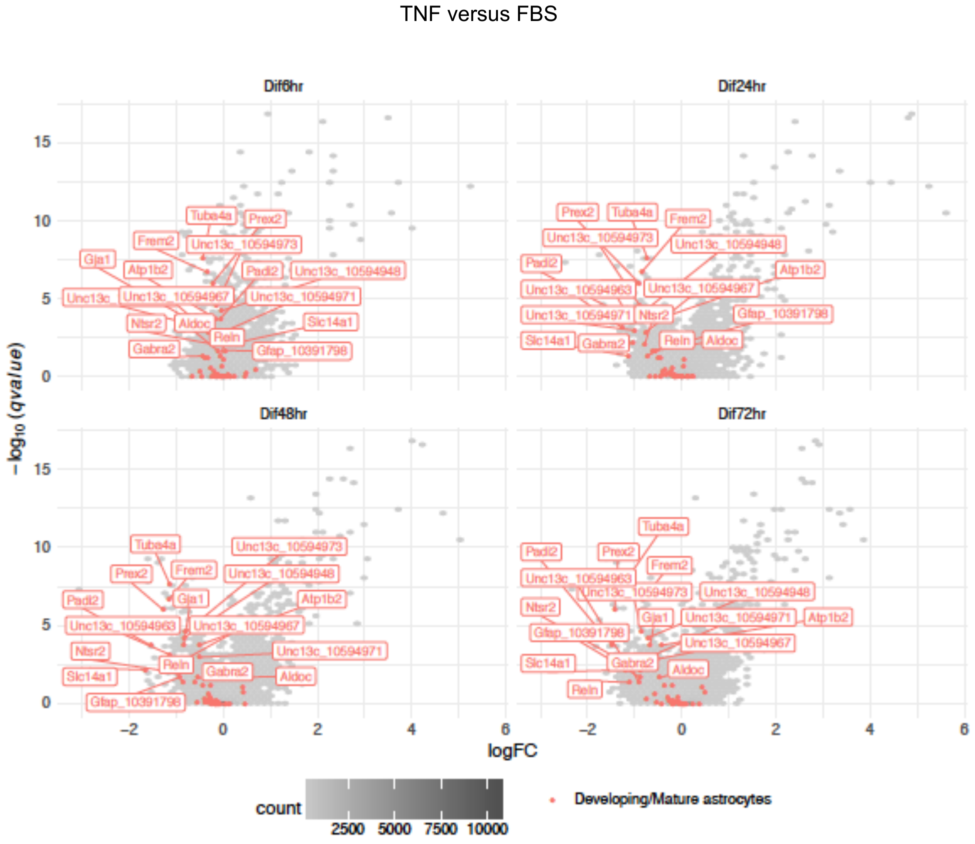

S3. Volcano plots for the 4 contrast analyses. Scatter plots of the -$\log_{10}$(q value) in function of the $\log_2$ fold change of the TNF stimulation compared to the FBS. All genes are binned into 60 bins and respective counts are displayed according to the grey gradient in the legend. Genes belonging to either the Developing/Mature astrocytes or Reactive astrocytes pathways are highlighted as an extra colored dot payer. The list was realized after a compilation of the publication by Cahoy et al.2008, Molofsky et al. 2012, Zhang et al. 2016 and the database from Network glia (http://www.networkglia.eu/en/astrocyte). Gene symbols for those pathways are displayed only if their *q-value* is < 0.05 (1.30103 as -$\log_{10}$). Genes with more than 1 probeset have the probeset id appended to their gene symbol. For example, *Unc13c* has 7 probeset ids and 10594971 is one probeset with a *q-value* < 0.05.

gene_symbol	q_value	pathways	Dif6hr	Dif24hr	Dif48hr	Dif72hr
Aldoc	0,023195509	Developing/Mature astrocytes	-0,108484194	-0,623772411	-0,547190474	-0,472335783
Atp1b2	1,83E-04	Developing/Mature astrocytes	-0,165389993	-0,444164308	-0,507112606	-0,424009581
Cd109	0,999999963	Reactive astrocytes	0,11323992	0,057332967	-0,073162425	-0,12913325
Cd14	6,47E-09	Reactive astrocytes	0,952756851	1,330993718	1,31578106	1,556442661
Cd44	0,839094887	Reactive astrocytes	0,594023698	0,380250744	0,129470256	-0,097077418
Col1a1	0,999999963	Developing/Mature astrocytes	-0,086822048	0,237944192	-0,113957697	0,063282724
Cxcl10	2,51E-17	Reactive astrocytes	3,568915223	4,801955216	4,192875129	2,877559571
Dusp14	0,999999963	Developing/Mature astrocytes	-0,07455089	-0,102142578	-0,161438615	-0,193615697
Emp1_10542355	0,946762837	Developing/Mature astrocytes	0,463650289	0,147685468	-0,382328077	-0,174503339
Emp1_10542355	0,946762837	Reactive astrocytes	0,463650289	0,147685468	-0,382328077	-0,174503339
Epha5	0,516860114	Developing/Mature astrocytes	-0,493108044	-0,403620425	-0,397310234	-0,749095731
Fbln5	0,999999963	Reactive astrocytes	0,029605152	0,059593798	-0,085957652	-0,091806005
Fgfr3	0,999999963	Developing/Mature astrocytes	-0,052936426	-0,143982654	-0,251702461	-0,016595649
Frem2	2,03E-07	Developing/Mature astrocytes	-0,338654914	-0,850897417	-1,148976612	-0,826826465
Frmd3	0,999999963	Developing/Mature astrocytes	0,154966566	-0,016409864	-0,145523115	-0,427044569
Fzd3	0,274443906	Developing/Mature astrocytes	-0,266443594	-0,195926426	-0,318756268	-0,571534535
Gabra2	0,047436228	Developing/Mature astrocytes	-0,439741303	-1,129289114	-0,60450402	-0,90957834
Gfap_10391798	0,021276084	Developing/Mature astrocytes	0,039681579	-0,558800917	-0,930691328	-0,88292039
Gfap_10391798	0,021276084	Reactive astrocytes	0,039681579	-0,558800917	-0,930691328	-0,88292039
Gja1	1,96E-04	Developing/Mature astrocytes	-0,752338454	-1,103180902	-0,845013904	-0,677242995
Glul_10350753	0,198930992	Developing/Mature astrocytes	-0,024021602	0,040009454	0,429687265	0,494593776
Glul_10374453	0,09265359	Developing/Mature astrocytes	0,009618488	0,054502628	0,409513533	0,437311928
Gmpr	0,999999963	Developing/Mature astrocytes	-0,186798541	0,104878478	0,039973891	0,021498544
Gstm7	0,999999963	Developing/Mature astrocytes	0,048628928	0,107813828	0,4670492	0,234856507
Icam1	0,929366505	Reactive astrocytes	-0,174991801	0,10825111	-0,282214442	0,12217262
Igfbp3	0,8393156	Reactive astrocytes	0,370033894	0,847058014	1,002397817	0,385455396
Kcnj10	0,066859121	Developing/Mature astrocytes	-0,382197045	-0,494236343	-0,434509691	-0,363093231
Lcn2	3,46E-11	Reactive astrocytes	3,628806392	5,677163393	5,026584035	3,8704022
Nes	0,999999963	Developing/Mature astrocytes	0,233914798	0,216687266	-0,09941952	-0,206515185
Npnt	0,929748851	Developing/Mature astrocytes	0,045843065	-0,452159351	-0,548772752	-0,536296748
Nsg2	0,9223006	Developing/Mature astrocytes	-0,456431724	-0,562635165	-0,201158034	-0,121452717
Ntsr2	0,008443307	Developing/Mature astrocytes	-0,21355212	-0,78207007	-1,2032149	-0,877545471
Osmr	0,002272316	Reactive astrocytes	1,48503663	0,772843115	0,251647703	0,078315577
Padi2	1,89E-04	Developing/Mature astrocytes	-0,058290586	-1,099136288	-1,540428933	-1,479488545
Prex2	9,86E-07	Developing/Mature astrocytes	-0,2237412	-0,906643533	-1,275639969	-1,425938936
Prkar2b	0,825920217	Developing/Mature astrocytes	-0,111068409	-0,411678258	-0,302372065	-0,041667048
Psmb8	3,08E-07	Reactive astrocytes	0,107290647	0,643844805	0,879011589	1,077695267
Ptx3	1,41E-07	Reactive astrocytes	2,297396096	0,676420733	0,148441408	0,022025132
Reln	0,046041569	Developing/Mature astrocytes	-0,067288725	-0,723659223	-0,856199171	-1,112090868
Sbno2	2,22E-04	Reactive astrocytes	1,064710669	0,655731052	0,265435503	0,286492368
Sema5b	0,999999963	Developing/Mature astrocytes	0,095619863	0,009761776	0,121515455	0,378171179
Sema6a	0,999999963	Developing/Mature astrocytes	-0,134943283	-0,126672327	-0,074411287	0,029221035
Serpina3n	0,769250263	Reactive astrocytes	1,388622609	1,105633488	-0,150457168	-0,299921643
Sertad4	0,839161609	Developing/Mature astrocytes	0,105530715	-0,154216604	-0,366085339	-0,085808549
Slc14a1	0,007065097	Developing/Mature astrocytes	0,265770253	-1,032900405	-1,650709433	-1,194332261
Slc1a3	0,999999963	Developing/Mature astrocytes	-0,035313669	-0,007936636	0,041972056	0,082646699
Sncaip	0,999999963	Developing/Mature astrocytes	-0,66264623	-0,684990191	-0,165283913	0,044700258
Socs2_10372069	0,624926189	Developing/Mature astrocytes	0,490909306	-0,271579894	-0,338149619	-0,178557393
Socs2_10394674	0,400889115	Developing/Mature astrocytes	0,683409648	-0,357672173	-0,280764612	-0,0760466
Sox9	0,999999963	Developing/Mature astrocytes	-0,240925432	-0,160121724	-0,169630381	0,011517347
Steap4	3,57E-13	Reactive astrocytes	3,662253704	4,497577306	3,683206899	3,601626521
Timp1	0,241937205	Reactive astrocytes	1,081626555	1,316150869	0,713994581	0,523350803
Tmeff2	0,999999963	Developing/Mature astrocytes	-0,021557006	-0,168125798	-0,122519575	-0,054032295
Tnfrsf21	0,434239626	Developing/Mature astrocytes	-0,193423366	-0,220575943	-0,29667231	-0,29146013
Tspan6	0,918068842	Developing/Mature astrocytes	-0,01930807	-0,078114691	-0,104875452	-0,209567005
Tuba4a	2,31E-08	Developing/Mature astrocytes	-0,430536844	-0,733299706	-1,135862337	-1,239616312
Unc13c_10594948	6,08E-05	Developing/Mature astrocytes	-0,044457075	-0,785974933	-0,828782454	-0,713779887
Unc13c_10594963	7,35E-04	Developing/Mature astrocytes	-0,409568362	-1,268369942	-1,137256639	-1,293493653
Unc13c_10594965	0,757645166	Developing/Mature astrocytes	-0,13683973	-0,227059214	-0,226650187	-0,271133246
Unc13c_10594967	0,001442012	Developing/Mature astrocytes	-0,320622726	-0,762487026	-0,79814981	-0,519198854
Unc13c_10594969	0,744546005	Developing/Mature astrocytes	-0,147024379	-0,336111881	-0,150082272	-0,23096728
Unc13c_10594971	0,001096841	Developing/Mature astrocytes	0,079094975	-0,999198679	-0,502218064	-0,700149674
Unc13c_10594973	2,74E-05	Developing/Mature astrocytes	-0,147830462	-0,8926765	-0,80546068	-0,858392972
Wnt5a	0,063961884	Developing/Mature astrocytes	-0,325981588	-0,456145879	-0,272639188	-0,196401618

S4. Genes belonging to either the Developing/Mature astrocytes or Reactive astrocytes pathways. The list was realized after a compilation of the publication by Cahoy et al.2008, Molofsky et al. 2012, Zhang et al. 2016 and the database from Network glia (http://www.networkglia.eu/en/astrocyte).

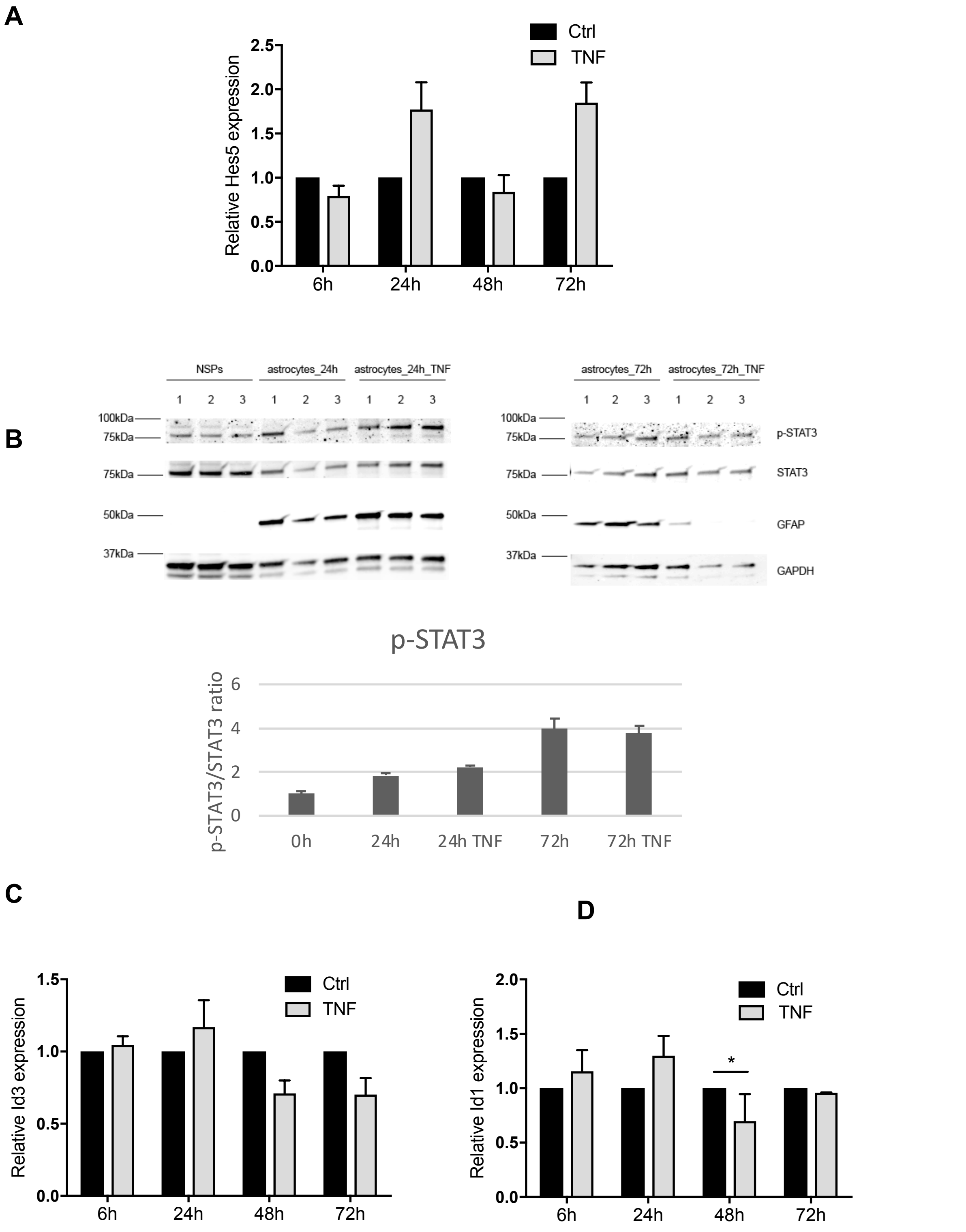

S5. Effect of TNF treatment on Hes5 (A), Id1 (C) and Id3 (D) genes expression obtained by RT-PCR. Each time point is normalized to its FBS control (=1). Results are given as mean ± SEM (n=4). B: Immunoblots from TNF treated and untreated at 0, 24h and 72h of differentiation. p-STAT3 protein expression is not affected by TNF.
